# New Year Special Issue, Animals in 2023

**DOI:** 10.3390/ani13010179

**Published:** 2023-01-03

**Authors:** Clive J. C. Phillips

**Affiliations:** 1 Institute of Veterinary Medicine and Animal Sciences, Estonian University of Life Sciences, Kreutzwaldi 1, 51014 Tartu, Estonia; clive.phillips@curtin.edu.au; 2 Curtin University Sustainability Policy (CUSP) Institute, Kent St., Bentley 6102, Australia

The management of animals on our planet is being scrutinised as never before. We face unprecedented loss of our animal species, from which it will take millions of years to recover, depending on how much damage is done before we start to reverse the disastrous mass extinction we have engineered. As well as the extinctions, the anthropogenic pressure on wildlife is depleting populations and causing them widespread stress, which depletes their immune system and makes them prone to new diseases. The human population has just come through a bad pandemic, which originated in wildlife under pressure from humans, in this case bats. Some of the blame must lie in the encroachment of humans into the territory of bats. Expansion of tourism into bat caves, widespread use of pesticides that accumulate in bats, demolition of old buildings providing roosting sites and the harvesting of bats for human consumption are all evidence of the pressure that bats are under in China and elsewhere [1]. Wildlife also suffers from widespread deforestation that forces them to search for novel feed sources, with plants grown for human consumption being a major target. Humans inevitably try to protect their food supplies, shooting the wildlife, scaring them away or netting the crops, which further stresses the animals. Further pandemics, conceivably much worse than COVID-19, are inevitable if we continue to flagrantly ignore the needs of our wildlife. Not only that, we are losing our ability to counter infections we might get from them with antimicrobials, in part because we’ve used them in a profligate manner to make small improvements in the efficiency of the growth of livestock, sterilising their gastrointestinal tracts (GIT) to ensure that every joule of energy, every gramme of protein is used to produce a saleable meat product, rather than feeding the microbiome that normally inhabits a healthy GIT. Too soon microbes develop ways to escape detection and slaughter by the antimicrobials; too late we are learning that a healthy microbiome is essential for animals’ disease resistance, wellbeing and safe feed digestion. 

As well as the anthropogenic assault, wildlife needs to cope with rapid climate change, with few resources being devoted to assisting them. Their opportunities for migration to climatic zones for which they evolved are extremely limited, as many wildlife species are confined to small pockets of indigenous fauna in the midst of intensive agriculture to feed humans. Livestock too face the ravages of climate change, which reduces their growth and milk production, and again causes stress: stress of escaping floods and fires, stress from depleted feed resources and restricted water supplies. 

Nowhere is the frantic attempt by humans to preserve their food resources more evident than in the livestock sector. Rapidly increasing demand for meat is evident as a result of the growing world population of humans and the greater affluence in parts of the world where meat consumption was hitherto uncommon. As human affluence grows, the affluence of other (non-human) animals shrinks alarmingly – their resources are squandered for our benefit. With limited land and labour resources, increasingly intensive animal production systems are being developed, using precision livestock farming to substitute for human care. The most extreme development in this field is the quest for a commercially available lab grown meat. In 2023 we will see new laboratories established for meat culture, with meat companies investing in the products, and perhaps one or more of the food companies offering their first artificial meat product to the market. 

It is clear from these growing challenges to the animals with which we share this planet, that we urgently need a strategy to manage all animals on planet earth more sustainably. To develop such a strategy, we need good scientific information, and that’s where *Animals* is becoming a force for good. Publications in *Animals* are mostly, although not uniquely, about improving the sustainability of our management of animals or predicting the effects of the current threats to our animals. For example, in 2021, Amira Gomez and I published a paper [2] foreshadowing a reduction in milk availability per person in Egypt from 61 kg/person/year in 2011 to 26 kg/person/year in 2064, as a result of population growth and increasing ambient temperatures. Such dramatic changes will require extensive planning to avoid major impact on the human population. Governments cannot ignore scientific evidence, or if they do they risk losing public support. We urgently need more evidence of the impact of these growing pressures on both human and animal populations, if we are to take the drastic actions necessary. These actions must change our way of farming, change our diet and change our attitudes to animals, if they, and we, are to see our way into the next century. Plan your contribution now, because everyone is needed to address these problems, not just scientists, not just politicians, nor even activists, but everyone united together to challenge the status quo and bring about the necessary changes. We have to consume less, much less: less animal products, less of the planet’s resources, and less of the genetic material that evolved to produce life on earth over billions of years. And we have to work quickly to restore the resources we have already squandered. We have to confront the vested interests that stand to benefit by maintaining the status quo. And above all else, although many of the difficulties faced recently have diminished our financial resources, we have to invest more to develop new technologies to address these issues. Innovative thinking is required to find solutions. Perhaps we need an annual per capita carbon quota, on a national or supranational basis, so that if people really must drive a vehicle emitting carbon dioxide, then they cannot consume much meat, that has a large carbon footprint. Perhaps we need a tax to preserve biodiversity, to address climate change and to restore degraded habitats. 

Eleven years ago, I strove to make scientific information on animal issues available to all, through our new open access journal, *Animals*. Now that we have achieve that, our next challenge is to make sure it is used effectively to address the most serious challenges to animal life on our planet, including humans, that we have ever faced. About 200 articles were published in Animals in 2022 on the themes of biodiversity, climate change and intensification of animal production. What are you doing to address these challenges?

This special issue welcomes contributions, especially original articles and reviews.
